# Targeted Co-Delivery of Docetaxel and cMET siRNA for Treatment of Mucin1 Overexpressing Breast Cancer Cells

**DOI:** 10.15171/apb.2018.045

**Published:** 2018-08-29

**Authors:** Naime Majidi Zolbanin, Reza Jafari, Jafar Majidi, Fatemeh Atyabi, Mehdi Yousefi, Farhad Jadidi-Niaragh, Leili Aghebati-Maleki, Dariush Shanehbandi, Mohammad-Sadegh Soltani Zangbar, Alireza Mohajjel Nayebi

**Affiliations:** ^1^Drug Applied Research Center, Tabriz University of Medical Sciences, Tabriz, Iran.; ^2^Pharmacology and Toxicology Department, School of Pharmacy, Tabriz University of Medical Sciences, Tabriz, Iran.; ^3^Department of Immunology, Faculty of Medicine, Mashhad University of Medical Sciences, Mashhad, Iran.; ^4^Immunology Research Center, Inflammation and Inflammatory Diseases Division, School of Medicine, Mashhad University of Medical Sciences, Mashhad, Iran.; ^5^Immunology Research Center, Tabriz University of Medical Sciences, Tabriz, Iran.; ^6^Department of Immunology, School of Medicine, Tabriz University of Medical Sciences, Tabriz, Iran.; ^7^Department of Pharmaceutics, Faculty of Pharmacy, Tehran University of Medical Sciences, Tehran, Iran.; ^8^Nanotechnology Research Centre, Faculty of Pharmacy, Tehran University of Medical Sciences, Tehran, Iran.

**Keywords:** Aptamer, Chitosan, cMET siRNA, Docetaxel, Metastatic breast cancer

## Abstract

***Purpose:*** Targeted treatment of breast cancer through combination of chemotherapeutic agents and siRNA had been drawing much attention in recent researches. This study was carried out to evaluate mucin1 aptamer-conjugated chitosan nanoparticles containing docetaxel and cMET siRNA on SKBR3 cells.

***Methods:*** Nano-drugs were characterized by transmission electron microscope, Zetasizer and loading efficiency calculation. siRNA entrapment onto nanoparticles, stability of siRNA-loaded nanoparticles and conjugation of mucin1 aptamer to nanoparticles were evaluated via separate electrophoresis. Cellular uptake of the targeted nanoparticles was evaluated through GFP-plasmid expression in mucin1+ SKBR3 vs. mucin1- CHO cells. Protein expression, cell viability and gene expression were assessed by Western Blotting, MTT assay, and Quantitative Real Time-PCR, respectively.

***Results:*** Characterization of nano-drugs represented the ideal size (110.5± 3.9 nm), zeta potential (11.6± 0.8 mV), and loading efficiency of 90.7% and 88.3% for siRNA and docetaxel, respectively. Different gel electrophoresis affirmed the conjugation of aptamers to nanoparticles and entrapment of siRNA onto nanoparticles. Increased cellular uptake of aptamer-conjugated nanoparticles was confirmed by GFP expression. cMET gene silencing was confirmed by Western Blotting. The significant (p ≤0.0001) impact of combination targeted therapy vs. control on cell viability was shown. Results of Quantitative Real Time-PCR represented a remarkably decreased (p ≤0.0001) expression of the studied genes involving in tumorigenicity, metastasis, invasion, and angiogenesis (STAT3, IL8, MMP2, MMP9, and VEGF) by targeted combination treatment vs. control.

***Conclusion:*** The mucin1 aptamer-conjugated chitosan nanoparticles, containing docetaxel and cMET siRNA, is suggested for treatment of mucin1^+^ metastatic breast cancer cells. However, further studies should be conducted on animal models.

## Introduction

Breast cancer treatment has become a complicated issue worldwide since the adverse reactions of the used medications decrease the patient compliance and the new drug resistance mechanisms are appeared.

cMET, the essential receptor tyrosine kinase for embryonic development of epithelial and endothelial cells, is normally activated by its ligand, Hepatocyte Growth Factor (HGF). The formed complex activates many signaling pathways such as Signal Transducer and Activator of Transcription 3 (STAT3). This causes an increase in inflammation, migration/ invasion, and angiogenesis via up-regulation of IL8, Matrix Metalloproteinases 2 and 9 (MMP2, MMP9), and Vascular Endothelial Growth Factor (VEGF), respectively. In many cancers, overexpression/ activation of cMET occur, which may be responsible for tumorigenicity, metastasis, and immunosuppression.^1,2^ HGF/ cMET signaling is increased in breast cancer cells, especially in the Her2^+^ subtype. This is related with drug resistances and reduced survival, and thus, cMET has become a therapeutic target.^3^ Standard approved therapeutic protocol for Her2^+^ metastatic breast cancer includes Trastuzumab, accompanied with the preferable chemotherapeutic agents, Docetaxel or Paclitaxel.^4^ Apart from these two mentioned common therapies, gene silencing has become a research interest. This can be put to use by applying micro RNA (miRNA) and small-interfering RNA (siRNA).^5^ Among the mentioned chemotherapeutic agents, docetaxel has been used successfully. However, its adverse hematological (febrile neutropenia) and non-hematological (hypersensitivity reactions, fluid retention, nail toxicities, etc.) reactions have reduced patients' compliance.^6^ The application of safe biodegradable nano-carriers, such as chitosan nanoparticles (NPs), is one of the novel solutions of pharmaceutical technology in reducing the adverse drug reactions. This is done for the delivery of chemotherapeutic agents, which results in dose reduction of loaded agent.^7^ On the other hand, using positively charged chitosan for loading the desired drug gives chance to load negatively charged easily degradable siRNA molecules simultaneously.^8^ Moreover, the mentioned co-delivery of chemotherapeutic drug and siRNA can be better targeted and specific to cancer cells through the novel DNA/RNA molecules, Aptamers (APT).^9^ The anti-mucin1 aptamer is used because mucin1 is one of the distinctly over expressed glycoproteins on breast cancer cells.^10^

The aim of this study was designing of the co-delivery system of docetaxel and cMET siRNA in the form of targeted chitosan NPs conjugated to the mucin1 APT. Cellular evaluations of the designed system were done on metastatic breast cancer cell line, SKBR3.

## Materials and Methods

### Materials

Carboxymethyl dextran (CMD), 1-Ethyl-3-(3-dimethylaminopropyl) carbodiimide (EDC), N-Hydroxysuccinimide (NHS) and 3-(4,5-dimethylthiazol-2-yl)-2,5-diphenyltetrazolium bromide (MTT) powder were all supplied from Merck^®^ (Darmstadt, Germany). Mucin1 aptamer (APT) with the following sequence (5' -Amino-C6-GGG AGA CAA GAA TAA ACG CTC AAG AAG TGA AAA TGA CAG AAC ACA ACA TTC GAC AGG AGG CTC ACA ACA GGC- 3') was purchased from TAG Copenhagen^®^(Frederiksberg, Denmark). Docetaxel (DTX) was ordered from AqVida^®^ (Hamburg, Germany). Human-specific cMET-siRNA (catalog number: sc-29397) and non-targeting (scrambled) siRNA (catalog number: sc-37007, Sense: UUCUCCGAACGUGUCACGUTT, Antisense: ACGUGACACGUUCGGAGAATT) were purchased fromSanta Cruz Biotechnology^®^ (Santa Cruz, CA, USA). The SKBR3 and CHO cell lines were obtained from the National Cell Bank of Iran (Pasteur Institute of Iran, Tehran, Iran). Roswell Park Memorial Institute (RPMI) 1640 medium, Fetal Bovine Serum (FBS), Trypsin, and Penicillin-Streptomycin were all purchased from Gibco^®^ (Waltham, MA USA). RNA extraction solution was purchased from SinaClon^®^ (Tehran, Iran). GFP containing plasmid was obtained from Clontech Laboratories (** **Mountain View, CA, USA). cMET primary monoclonal antibody (catalog number: sc-514148), horseradish conjugated anti-IgG secondary polyclonal antibody (catalog number: sc-2005), and beta-actin primary monoclonal antibody (catalog number: sc-47778) were all purchased from Santa Cruz Biotechnology^®^ (Santa Cruz, CA, USA).

#### Depolymerization of 50 kDa chitosan

In order to prepare chitosan with MW of 50 kDa, the chitosan with medium MW of 400 kDa was depolymerized according to the protocol described previously.^11^ Briefly, 2 g of chitosan was dissolved in 10 ml of acetic acid (6% v/v) to gain 2% v/v solution of chitosan in acetic acid. Thereafter, 10 ml of sodium nitrite (NaNO_2_, 2.5 mg/ml) was added to the dissolved chitosan under magnetic stirring at 25 °C for 2 h in order to obtain 50 kDa chitosan. In order to precipitate the depolymerized chitosan (dCS), the pH was gained to 9 by adding of NaOH (4M). The precipitated depolymerized chitosan (white/ yellow in color) was then filtrated and washed for three times with acetone. The yield product was dissolved in 40 ml of acetic acid (0.1 N). The dialysis of the dissolved chitosan in acetic acid against demineralized water was done to obtain the purified depolymerized chitosan (Sigma dialysis tubes, cutoff 12 kDa). It was then lyophilized by Alpha 2-4 LD plus freeze dryer (Martin Christ, Osterode am Harz, Germany). The MW of the yield product was assessed via gel permeation chromatography (Agilent Technologies, Santa Clara, CA, USA), as described previously.^12^

#### Preparation of loaded nanoparticles

Nanodrugs were prepared through ionotropic gelation method. dCS (0.1% w/v) was dissolved in diethylpyrocarbonate (DEPC) treated water under magnetic stirring (140 rpm) for two hours. 3 µl of siRNA (19 µg/µl, 3×10^-1^ µmol) and 5 µl of DTX (50 µg/ml, 61.9 µM) were added to 1.2 ml of CMD (0.1% w/v). Then the obtained mixture was added dropwise (with time intervals of 5 seconds between drops) to 1 ml of dCS solution under gentle stirring (140 rpm) for 10 minutes and then incubated in the dark place for 30 minutes at 25 °C. In order to remove the unloaded drugs, membrane dialysis bag (12 kDa cutoff, Merck^®^, Darmstadt, Germany) was used twice in DEPC-treated water for 90 minutes and once overnight.

#### In vitro evaluations of nanoparticles

Investigation of shape and surface morphology of freshly prepared NPs were assessed by transmission electron microscope (TEM, LE-O906, Zeiss^®^, Jena, Germany). Particle size, Polydispersity Index (PDI), and zeta potential were determined by Photon Correlation Spectroscopy using Zetasizer Nano-ZS (Malvern Instruments, Malvern, UK) at a wavelength of 633 nm.

In order to confirm siRNA entrapment onto nanoparticles, the electrophoresis on a 4% agarose gel was done with siRNA loaded NPs, blank NPs, and plain siRNA.

The UV-vis spectrophotometer (Nanodrop^®^ 2000, Thermo Fisher Scientific, Waltham, MA, USA) was used for measuring loading efficiency % (LE%) of cMET siRNA (at 260 nm) and DTX (at 230 nm). Ultimately, the LE% was calculated with the help of the following equation:

$$\text{LE}\left(\%\right)=\left[1-\left(\frac{\text{OD of sample in supernatant}}{\text{OD of initial feeding amount of sample}}\right)\right]\times 100$$

#### Serum and heparin stability of siRNA loaded nanoparticles

400 µl of the siRNA-loaded NPs (0.15 µg/µl) was added to 200 µl of FBS (10%) and the mixture was incubated at 37 °C shaker for sequential sampling at time intervals of 0, 2, 8, 12, 24, and 48 hours. The collected samples were kept at -20 °C. Furthermore, 60 µl of siRNA-loaded NPs was added to heparin solution (2 µg/µl) in volumes of 0, 0.6, 1.5, and 3 µl and they all were incubated at 37 °C shaker for 1 h. Moreover, 4% agarose gel electrophoresis was run with the mentioned samples and the plain siRNA as control.

#### In vitro DTX and siRNA release

The release of siRNA loaded and DTX loaded NPs was evaluated by incubation of the mentioned samples inside the membrane dialysis bag (12 kD cut off, Merck) in 50 ml of phosphate buffer solutions (PBS, pH = 7.4 and pH = 5.5) at 37 °C for 120 h. Subsequently, 2 ml of solutions was withdrawn and replaced with the same volume of fresh PBS under same condition at several intervals. Finally, siRNA and DTX released contents were assessed by UV-vis spectrophotometer at 260 and 230 nm, respectively. Furthermore, the released medium collected from blank NPs was used as the blank sample. In vitro siRNA and DTX release (%) were calculated with the help of the following equation:

$$\text{Released DTX or siRNA (\%) = }\left(\frac{\text{OD of DTX or siRNA in the PBS}}{\text{OD of initial total content of DTX or siRNA}}\right)\times 100$$

#### Conjugation of mucin1 aptamer to chitosan nanoparticles

NPs were suspended in 200 µl of DNase/RNase-free water and activated with EDC (10 mg) and NHS (8 mg) and stirred at 25 °C for 2 h in order to conjugate APT to NPs, according to the previously described EDC/NHS method.^13^ The unreacted EDC and NHS were removed through membrane dialysis bag (12 kDa cutoff, Merck^®^, Darmstadt, Germany). Then, 5'-NH_2_-modified Mucin1 APT (1% w/ w) was reacted with activated NPs for 8 h at 25 °C. Eventually, in order to remove un-conjugated APT, the sample was centrifuged (2 × 10 min, 16,000 g, 5 °C) and washed with DEPC-treated water. Agarose gel (4%) electrophoresis was run in Tris-acetate-EDTA (1M) solution to confirm APT conjugation to NPs. To calculate APT conjugation efficiency, the Optical Density (OD) of the final sample was measured at 260 nm via UV-vis spectrophotometer (Nanodrop^®^ 2000).

#### Evaluation of cellular uptake

In order to compare efficiency of mucin1 APT on cellular uptake of NPs, SKBR_3_ cells (mucin1^+^) and CHO cells (mucin1-) were seeded in 6-well culture plates (1.5 × 10^5^ cells/ well) with RPMI-1640 medium supplemented with FBS 10%, penicillin 100 units/ mL and streptomycin 100 µg/ mL, kept at 37 °C in 5% CO_2_ incubator. To prepare the pharmaceutical groups, briefly, CMD solution (0.1% w/ v) was prepared by dissolving CMD in DEPC-treated water (pH = 7). Subsequently, 3 µl of GFP plasmid (1 µg/ µl) was added into 1.2 ml of CMD. Eventually, the yielded aqueous solution was added dropwise into 1 ml of dCS solution under gentle stirring (140 rpm) for 10 minutes. The nanodrugs were then incubated in the dark at room temperature for 30 minutes. Aptamer conjugation was done according to the EDC/ NHS method described at the previous section. Then the cells were treated with "NPs + GFP" and "NPs + APT + GFP" for 24 h and incubated at 37 °C in 5% CO_2_. Thereafter, the cells were washed with PBS (pH = 7.4) and fixed with 4% formaldehyde for 30 minutes at 25 °C. The fixed cells were incubated with DAPI (nucleic acid stain) for 5 minutes. Cellular uptake was assessed by cell imaging system, Cytation^TM^5 (BioTekInstruments, Winooski, Vermont, USA).

#### Protein extraction and western blotting

5 × 10^6^ SKBR3 cells were treated by "NPs + siRNA" for 48 h. The protein extraction by RIPA lysis buffer system (Santa Cruz Biotechnology^®^, Santa Cruz, CA, USA) and the protein concentration assay via Bradford method (Protein assay Kit, Razibiotech, Tehran, Iran) were done. The supernatant was stored at -80 °C. Thereafter, the electrophoresis was run on 8% SDS-PAGE gel and the wet transfer system (Bio Rad Laboratories, Hercules, CA, USA) was used for transfection of proteins onto PVDF membrane. The membrane was blocked via incubation at 4 °C for overnight in Tris-Buffered Saline Tween 20 (TBST) buffer supplemented by 3% Bovine Serum Albumin (BSA). Subsequently, incubation at 25 °C for 1 h with 1:500 dilution of cMET primary antibody was done. The 1:2000 dilution of horseradish conjugated anti-IgG secondary antibody was added for membrane washing and was incubated for 2 h at 25 °C. The monoclonal beta-actin primary antibody at a dilution of 1:500 was used as the control. Super Signal West Pico Chemiluminescent Substrate (Thermo fisher scientific, Waltham, MA, USA) was used to detect the created bands. Protein bands' intensity were quantified by ImageJ software (National institutes of health, Maryland, USA) and normalized to the corresponding beta-actin.

#### MTT bioassay

The cytotoxicity and IC50-values of free DTX and NPs loaded by DTX were assessed previously.^14,15^ To evaluate cell viability, the SKBR3 cells were seeded in 96-well plates (1 × 10^4^ cells/well) in triplicate mode and treated for 24 and 48 hours. Then, 100 µl of fresh medium and 100 µl of MTT solution (5mg/ ml) were added to each well and incubated at 37 °C for 4 h. After removing the medium, 200 µl of dimethyl sulfoxide (DMSO) and 25 µl of Sorenson's buffer were added and incubated (20 minutes, 25 °C). Optical density was read at 570 nm versus 630 nm reference wavelength by ELISA reader (Stat Fax 2100, Awareness Technology, Palm City, FL, USA). Ultimately the cell viability (%) was calculated with the help of the following equation:

$$\text{Cell viability (\%) = }\left(\frac{\text{OD of sample}}{\text{OD of control}}\right)\times 100$$

#### RNA extraction, cDNA synthesis and Quantitative Real Time-PCR analysis

5 × 10^5^ SKBR3 cells/well were cultured and treated for 24 and 48 hours. RNA extraction was done by RNX-Plus^®^solution according to manufacturer’s instructions. The high-quality RNA samples which were assessed by UV-vis spectrophotometer at 260 nm, were stored at -70 °C. Thereafter 1 µl of the random hexamer, 1 µl of oligo dT, 4 µl of RT buffer (5X), 0.5 µl of RT and 2 µl of dNTP mix were added to 1 µl of total RNA (5 ng). In order to synthesis complementary DNA (cDNA), the reaction was carried out in a Bio-Rad thermal cycler (Hercules, CA, USA) at 25 °C for 5 min, 42 °C for 60 min and 85 °C for 5 min. The QRT-PCR method was then conducted by using the SYBR Green Real-time PCR master mix (Ampliqon^®^, Odense, Denmark) with the total volume of 10 μL on the LightCycler® 96 System (Roche, Basel, Switzerland). The primers were designed by OLIGO 7 software (Table 1). The system was setup according to the following: denaturation (94 °C, 10 minutes), amplification (94 °C, 45 cycles of 10 seconds), annealing temperature (30 seconds) and extension (72 °C, 10 seconds). Melting curve analysis was used for gene-specific amplification. Determination of relative mRNA level was done through the 2^-ΔΔCt^ (Livak) method on the basis of relative expression of target mRNA to 18S rRNA mRNA level as the housekeeping gene.

#### Statistics

Statistical analysis was performed via GraphPad Prism 6.0. The results were evaluated by one-way ANOVA test and Tukey post-test as necessity. Probability values of less than 0.05 were considered significant. The results presented in text and tables represent mean± standard deviation (SD).

**Table 1:** Sequences of primers

**Primer**	**Sequence (5'→ 3')**	**Gene accession number**
**18S rRNA**	F: GATCAGATACCGTCGTAGTTCC	NR_146144.1
	R: CTGTCAATCCTGTCCGTGTC	
**STAT3**	F: AGTTTCTGGCCCCTTGGATTG	NM_139276.2
	R: CAGGAAGCGGCTATACTGCTG	
**IL8**	F: CGGAGCACTCCATAAGGCA	NM_000584.3
	R: TGGTCCACTCTCAATCACTC	
**MMP2**	F: GCCCTCCTGGGAATGAAGCAC	NM_001127891.2
	R: GCATTGCCTCTGGACAACACA	
**MMP9**	F: ATTCATCTTCCAAGGCCAATCC	NM_004994.2
	R: CTTGTCGCTGTCAAAGTTCG	
**VEGF**	F: TCACCAAGGCCAGCACATAG	NM_001025366.2
	R: GACAGCAGCGGGCACCAAC	

18S rRNA: 18S ribosomal ribonucleic acid, STAT3: Signal transducer and activator of transcription 3, IL8: Interleukin 8, MMP: Matrix metalloproteinase, VEGF: Vascular endothelial growth factor

## Results and Discussion

### Physicochemical characteristics of nanoparticles

**Figure 1:**
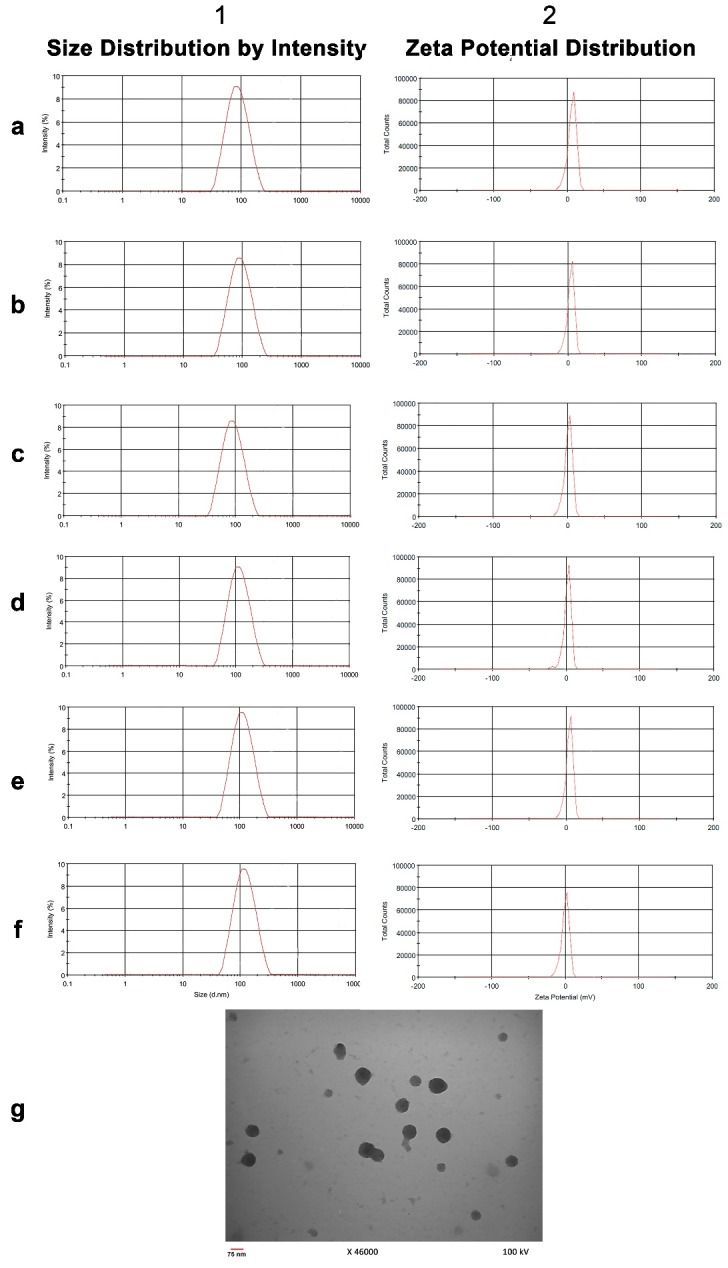
Particle size and Zeta potential distribution for (a) blank dCS, (b) dCS + APT, (c) dCS + siRNA, (d) dCS + APT + siRNA, (e) dCS + DTX+ siRNA and (f) dCS + APT + DTX + siRNA. g) Transition electron microscopy image of NPs. (dCS: depolymerized chitosan, APT: Aptamer, DTX: Docetaxel, NPs: Nanoparticles)

**Figure 2:**
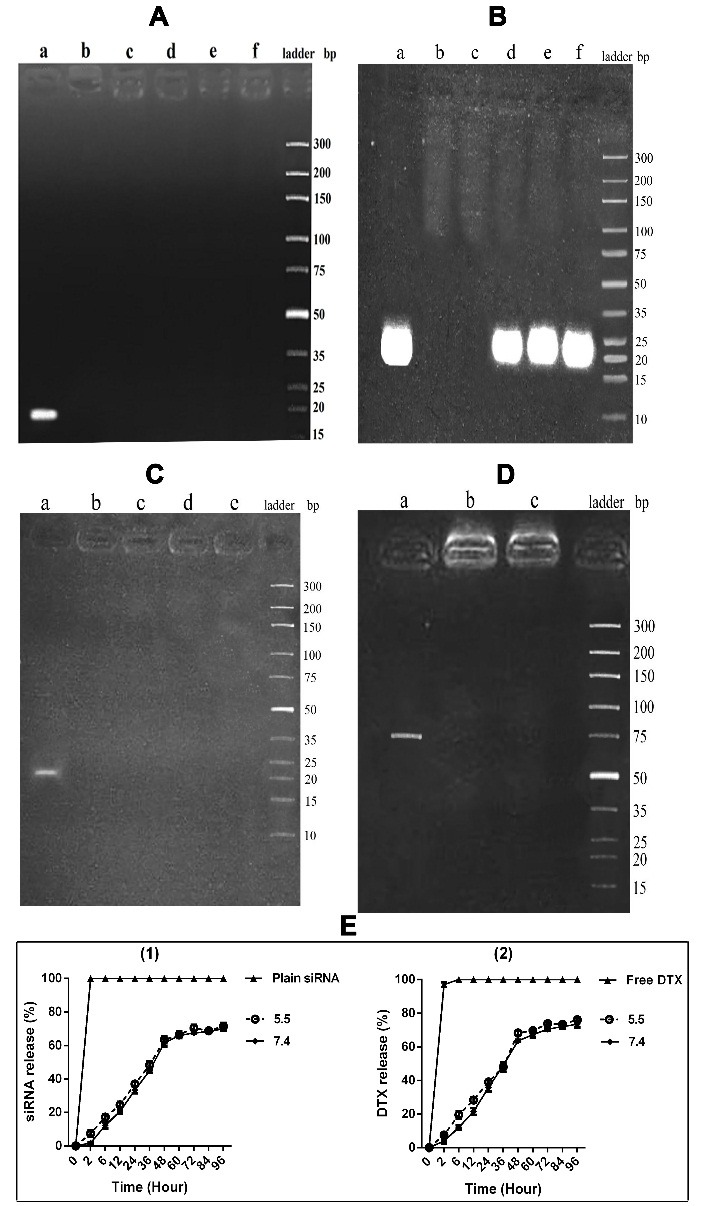
In vitro characterizations of nanodrugs. **A)** Electrophoresis of siRNA loaded NPs. a) Plain siRNA, b) NPs +siRNA (28.5 µg), c) NPs +siRNA (57 µg), d) NPs +siRNA (85.5 µg), e) Blank NPs, f) NPs+ APT+ siRNA (85.5 µg). **B)** FBS stability test. a) Plain siRNA, b) NPs +siRNA (2 h), c) (8 h), d) (12 h), e) (24 h), f) (48 h). **C)** Heparin stability test. a) Plain siRNA (57µg), b) NPs +siRNA, c) [NPs +siRNA] + 0.6 µl Heparin (2 µg/ml), d) [NPs +siRNA] + 1.5 µl Heparin, e) [NPs +siRNA] + 3 µl Heparin. **D)** Aptamer conjugation confirmation. a) Intact mucin1 APT, b) un-purified (NPs + APT), c) purified (NPs + APT). **E)** Drug release (%). a) siRNA release (%), b) DTX release (%) at PBS solutions with pH of 5.5 and 7.4; Equivalent free drugs (siRNA, DTX) were dispersed in PBS buffer as a control. (NPs: Nanoparticles, APT: Aptamer, DTX: Docetaxel)

The results of mean diameter size, zeta potential, and PDI are represented in Figure 1 and Table 2. The morphological property of the NPs containing DTX and siRNA, which was obtained through TEM, is shown in Figure 1. This represented the smooth and spherical shaped NPs. Figure 2-A indicated the electrophoresis of siRNA loaded NPs, which confirmed the complete entrapment of siRNA onto the NPs. The loading efficiency of siRNA loaded NPs and DTX loaded NPs was 90.7% and 88.3%, respectively. As results of characterizations of the NPs represented, all nano-drugs had a smooth spherical surface with a positive charge and the diameter less than 150 nm. According to other studies, the positive charge on NPs causes increased uptake and cytotoxicity in tumor cells.^16^ On the other hand, the larger size of NPs (that is more than 200 nm) would lead to more degradation by phagocytes.^17^ Moreover, as the positive charge increases, the possibility of nonspecific interactions with non-tumor cells increases too. Therefore, the importance of NP modification through specific APT utilization becomes more obvious. This consequently causes targeted delivery of drug to the specific site of action.^18^ Results of our study determined that the size, zeta potential, and pharmacokinetic properties of nano-drugs were not affected by APT conjugation. These results are consistent with the findings of Sayari et al.^13^ and Dhar et al.,^19^ which affirm the advantage of the APT to antibodies that have high impacts on the size and zeta potential. Another advantage of the appropriate positive charge of chitosan NPs is its usefulness for more efficient loading of therapeutic oligonucleotides such as siRNA.^20^ Furthermore, other investigations have reported that the molecular weight of chitosan plays a very important role on nanoparticles' size and loading efficiency of siRNA.^21^ On the basis of the study by Jadidi et al.,^22^ the most appropriate molecular weight of chitosan with the highest loading efficiency of oligonucleotides, is approximately 50 KDa.

**Table 2:** Results of mean diameter size, zeta potential and polydispersity index (PDI)

**Nano-drug**	**Characteristics**		
	**Mean diameter size (nm)**	**Zeta potential (mV)**	**PDI**
**Blank dCS**	93.1±5.1	18.9±0.2	0.212
**dCS+APT**	98.7±4.5	12.9±0.5	0.238
**dCS+siRNA**	95.2±2.7	12.8±0.3	0.218
**dCS+APT+siRNA**	105.8±1.3	11.1±0.1	0.281
**dCS+DTX+siRNA**	102.3± 6.7	13.2± 0.3	0.227
**dCS+APT+DTX+siRNA**	110.5± 3.9	11.6± 0.8	0.292

dCS: depolymerized chitosan, DTX: docetaxel, APT: aptamer

### Serum and heparin stability of siRNA loaded nanoparticles

As represented in Figure 2-B, releasing of siRNA from NPs was observed 12 h after incubation with FBS. Furthermore, the electrophoresis of siRNA-loaded NPs, which were incubated with different concentrations of heparin, represented the stable structure of NPs in this medium (Figure 2-C). Stability of the NPs in the *in vivo-*like environments, is one of the important factors which should be considered for drug delivery. Exposure of the NPs to the FBS and polyanions such as heparin is suitable for evaluation of stability of positively charged NPs in these negatively charged environments. In this regard, Jadidi et al.^22^ stated that heparin had no impact on siRNA loaded chitosan NPs and the siRNA releasing started after 9 hours of exposure to serum solution and completed at the 48^th^ hour. However, Raja et al.^23^ reported that chitosan NPs, which were loaded with siRNA, were stable in serum environment up to 48 hours.

### In vitro DTX and siRNA release

Figure 2-E indicated that siRNA was progressively released till 48 hours and the steady state phase started at the 60^th^ hour. The pattern of DTX release in both mentioned pH indicated that the gradual releasing continued up to 60 hours and the steady state phase started at the 72^nd^ hour. For the formulations in both pH, releasing of the encapsulated content of the NPs reached to 50% at the 36^th^ hour. The pH 7.4 and 5.5 simulated the pH of normal and tumoric tissues, respectively. According to the study by Jadidi et al.,^22^ releasing of the siRNA from chitosan-lactate NPs was gradually continued up to 72^nd^ hour and then reached to the steady state phase.

### Evaluations of mucin1 aptamer-conjugated nanoparticles

As represented in Figure 2-D, the agarose gel electrophoresis of intact APT was compared with the unpurified and purified conjugations of "NPs + APT". The obvious band shown with intact APT in comparison to other formulations confirmed the appropriate conjugation of APT to NPs. Results of Nanodrop^®^ measurement represented that 92.3% of APT was conjugated to NPs. Conjugation of mucin1 APT to NPs was through activation of connection sites on NPs via EDC and NHS that caused these sites' interaction with NH_2_ groups of the mucin1 APT. The agarose gel electrophoresis confirmed this conjugation. The mucin1 APT conjugated NPs created no distinct band on the agarose gel, neither before nor after purification. This finding showed that the activated NPs interacted with approximately all molecules of APT and no detectable free APT remained. Furthermore, utilization of mucin1 APT for targeted delivery increased the absorption of NPs to tumor cells. It seems that mucin1 APT plays an important role in uptake of the NPs by mucin1 expressing cells. However, the usage of mucin1 APT conjugated NPs on mucin1- cells did not change the level of cellular uptake. The studies by Ghasemi et al.^24^ and Sayari et al.^13^ confirmed the results of our study.

### Cellular uptake

**Figure 3:**
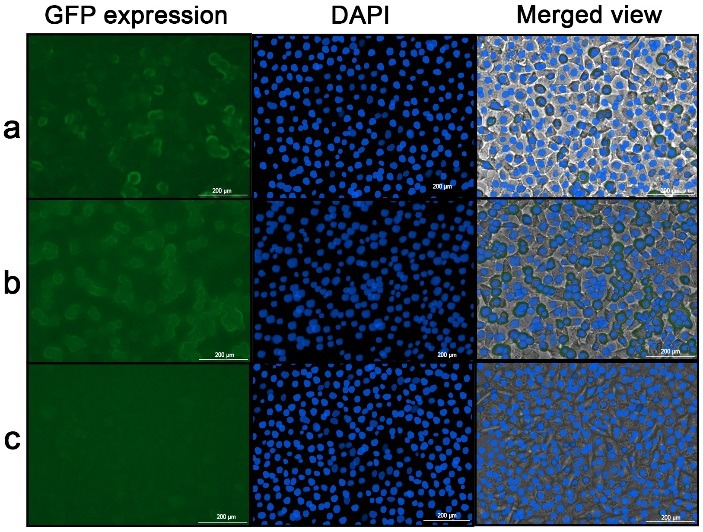
Cellular uptake evaluation. a) SKBR3 cells treated by NPs + GFP (1µg/µl); b) SKBR3 cells treated by NPs + GFP + APT and c) CHO cells treated by NPs + GFP + APT (GFP: Green Fluorescent Protein, NPs: Nanoparticles, APT: Aptamer)

The fluorescence intensity of GFP expression in cell was the qualitative indicator of cellular uptake of NPs. Figure 3 showed that the most fluorescence reaction occurred in SKBR3 cells that were treated with "NPs + GFP + APT" and the least was shown with CHO cells, treated by "NPs + GFP + APT". According to the study of Ghasemi et al.,^24^ the significant uptake of the mucin1 APT conjugated NPs by HT-29 mucin1^+^ cells was obvious in comparison to the non-targeted NPs. Actually, mucin1 APT acts like a linker between the NPs and the mucin1 expressing cells and facilitates cellular uptake of NPs.

### Evaluation of cMET protein expression

**Figure 4:**
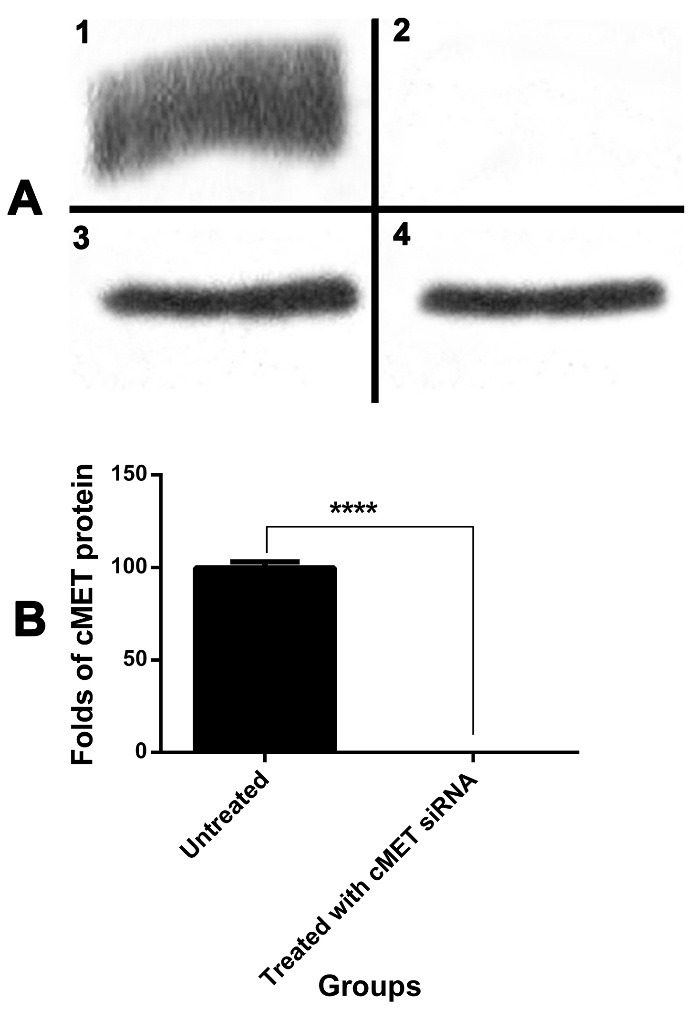
Protein expression assay. **A)** Western Blotting. 1) cMET protein expression in control SKBR3 cells; 2) cMET protein knock downing by siRNA in SKBR3 cells; 3) β-actin protein expression in control SKBR3 cells. 4) β-actin protein expression in SKBR3 cells treated with cMET siRNA. **B)** Quantification of cMET protein expression by ImageJ software; ****: *p*≤ 0.0001

For confirming cMET gene silencing via siRNA, western blotting assay was used. According to Figure 4, cMET protein was over expressed in untreated SKBR3 cells and its expression was silenced in the cells treated by "NPs + siRNA" for 48 h.

After cellular uptake of the NPs containing siRNA, the siRNA is released inside the cells. It targets the complementary mRNA in the cytoplasm and subsequently knock downs the targeted protein. Results of western blotting assay represented this knock downing of cMET protein after 48 h of treatment with siRNA-loaded NPs. 

### Cell viability assay

**Figure 5:**
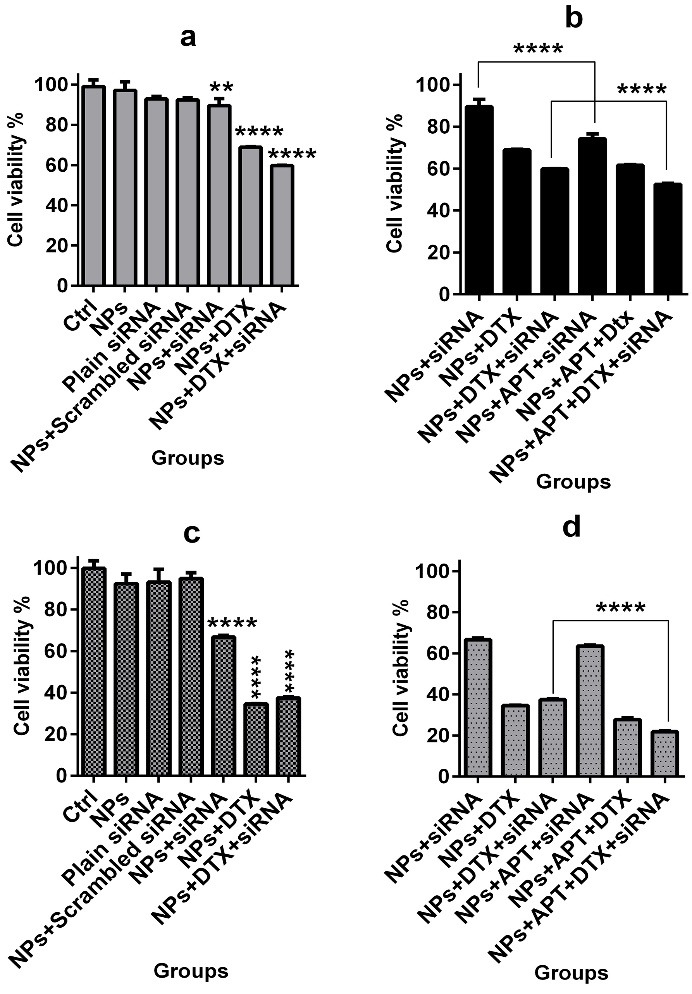
MTT assay. Control groups *vs.* groups lacking mucin1 APT (a: 24 h and c: 48 h); groups lacking mucin1 aptamer *vs.* groups containing mucin1 APT (b: 24 h and d: 48 h); **: *p*≤ 0.01, ****: *p*≤ 0.0001 (APT: Aptamer)

In order to investigate cell viability of SKBR3 cells in exposure to different treatment groups, MTT tetrazolium bioassay was done. As represented in Figure 5 (a and c), comparison of the treatment groups lacking mucin1 APT *vs.* control cells showed a significant decline in cell viability percentage in both 24 and 48 hours after treatment (*p* ≤0.0001 for all mentioned comparisons;* p* ≤0.01 for "NPs + siRNA" at 24 h). Also when comparing "NPs + siRNA" *vs.* "NPs + APT + siRNA" and "NPs + siRNA + DTX" *vs*. "NPs + APT + siRNA + DTX" at 24 h after treatment, the significant (*p* ≤0.0001) difference was obvious which showed the role of APT-conjugated NPs on cell viability (Figure 5-b). The significant (*p* ≤0.0001) difference represented when comparing "NPs + APT + siRNA + DTX" *vs.* "NPs + siRNA + DTX" at 48 h (Figure 5-d). Several investigations have been done on nano-formulating of DTX and evaluation of its impact on cellular viability and toxicity in various cancerous cell lines.^7,25^ According to our study, using of chitosan NPs for the delivery of DTX was safe since the intact NPs had no toxic effects on cell viability. Usage of the combination therapy system such as "DTX + siRNA" loaded NPs, as compared to monotherapy with "DTX"-loaded NPs, represented better results on inhibition of cell viability. Furthermore, providing the targeted therapy via APT resulted in improvement of the combination therapy. According to the results of cell viability after 48 h of treatments, when comparing "NPs + DTX" *vs.* "NPs + DTX + siRNA" and "NPs + APT + DTX" *vs.* "NPs + APT + DTX + siRNA", no significant difference was represented which showed that cMET siRNA did not have a significant effect on cell viability and also could not have a synergistic effect with DTX.

### Gene expression assay

**Figure 6:**
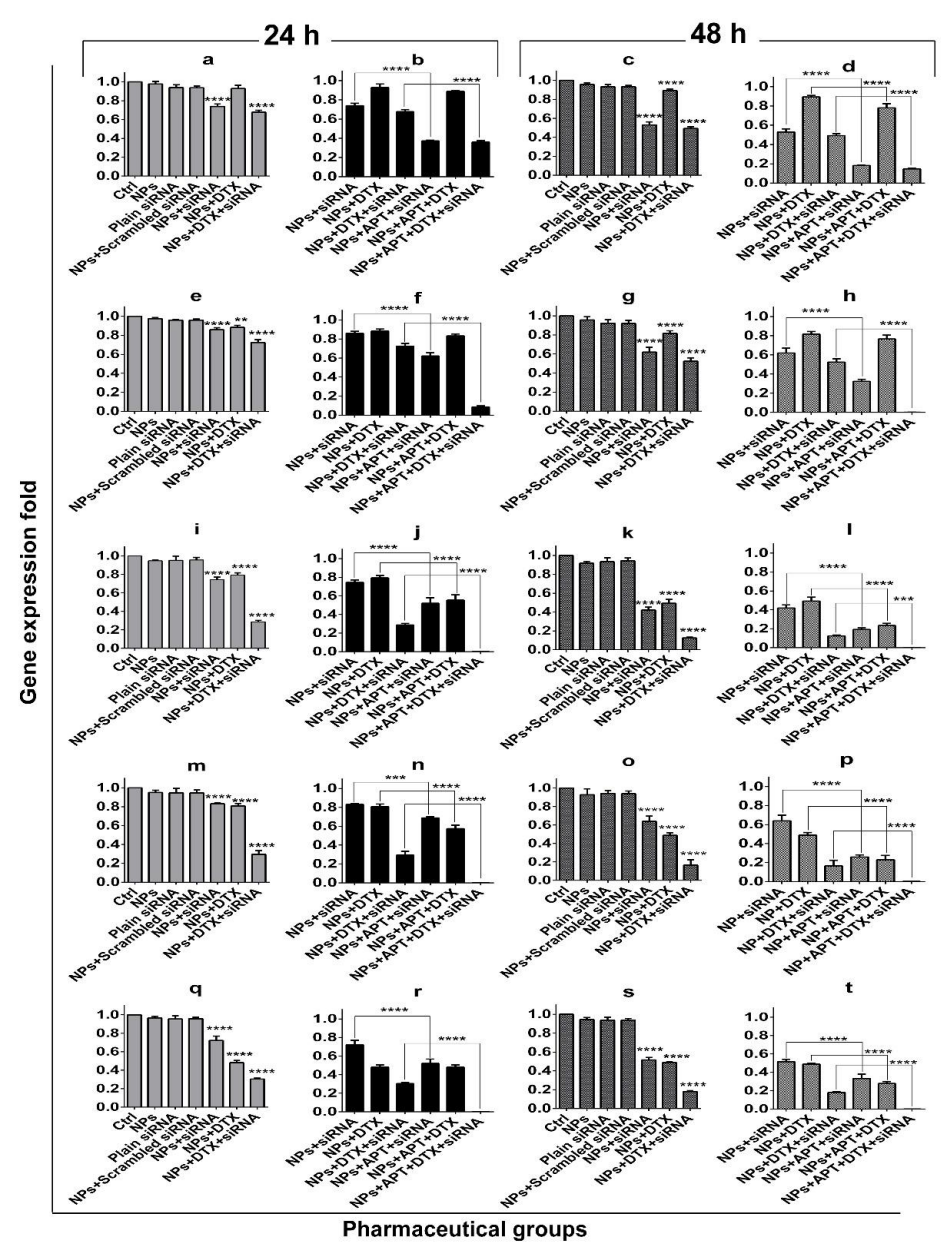
Gene expression folds at 24 h and 48 h for IL8 (a, b, c and d); STAT3 (e, f, g and h); MMP2 (I, j, k and l); MMP9 (m, n, o and p) and VEGF (q, r, s and t). **: *p*≤ 0.01, ***: *p*≤ 0.001 and ****: *p*≤ 0.0001.

Quantitative Real Time-PCR was done to assess the effect of different treatment groups on the expression of five genes including IL8, STAT3, MMP2, MMP9, and VEGF. All data is normalized to the housekeeping gene (18S rRNA). Figure 6 shows the related results gene by gene in two time intervals of treatment (24 h and 48 h).

About IL8 gene, comparison of the "NPs+ siRNA" and "NPs+ DTX+ siRNA" groups with the control group showed significant reduction (*p* ≤0.0001) of gene expression after 24 and 48 hours. However, "NPs+ DTX" group in compare to the control showed the significant reduction (*p* ≤0.0001) at 48 h (Figure 6, a and c). When comparing "NPs+ APT+ siRNA" *vs.* "NPs+ siRNA" and also "NPs + APT + DTX + siRNA" *vs.* "NPs + DTX + siRNA" the significant reduction (*p* ≤0.0001) in IL8 expression was obvious after 24 and 48 hours. The comparison of the "NPs+ APT+ DTX " group *vs.* "NPs + DTX" represented the significant reduction (*p* ≤0.0001) of IL8 expression at 48 h (Figure 6, b and d).

About the STAT3 gene, the comparison of the "NPs + siRNA" and "NPs + siRNA + DTX" groups *vs.* control group represented significant reduction (*p* ≤0.0001) of gene expression in both time intervals (Figure 6, e and g). Also, "NPs + DTX" *vs.* control significantly reduced gene expression at 24 h (*p* ≤0.01) and 48 h (*p* ≤0.0001). Comparison of the mucin1 APT containing groups with the lacking ones in both 24 h and 48 h, represented significant reduction (*p* ≤0.0001) of gene expression by "NPs + APT + siRNA" and "NPs + APT + DTX + siRNA" groups (Figure 6, f and h).

Gene expression patterns of MMP2 were as the following: all mucin1 APT lacking groups *vs.* control group (Figure 6, i and k) represented significant reduction (*p* ≤0.0001) of gene expression after 24 and 48 hours. Also, all mucin1 APT containing groups *vs.* mucin1 APT lacking corresponding groups (Figure 6, j and l) represented significant reduction (*p* ≤0.0001) of gene expression after 24 and 48 hours. However, comparison of the "NPs + APT + DTX + siRNA" group *vs.* "NPs + DTX + siRNA" group represented significant reduction (*p* ≤0.001) of the gene expression after 48 h.

About MMP9, the comparison of all treatment groups *vs.* control group represented significant reduction (*p* ≤0.0001) of gene expression at 24 and 48 hours (Figure 6, m and o). Furthermore, all mucin1 APT containing groups *vs.* mucin1 APT lacking corresponding groups (Figure 6, n and p) represented significant reduction (*p* ≤0.0001) of gene expression after 24 and 48 hours. However, the "NPs + APT + siRNA" group *vs.* "NPs + siRNA" significantly decreased (*p* ≤0.001) gene expression at 24 h.

About the VEGF gene, all mucin1 APT lacking groups *vs.* control group significantly decreased (*p* ≤0.0001) gene expression at 24 and 48 hours (Figure 6, q and s). Also, "NPs + APT + siRNA" and "NPs + APT + DTX + siRNA" groups *vs.* APT lacking corresponding groups showed significant reduction (*p* ≤0.0001) of gene expression at 24 and 48 hours. However, the "NP + APT+ DTX" group *vs.* "NP + DTX" represented the significant reduction (*p* ≤0.0001) of gene expression after 48 h (Figure 6, r and t).

It has been proved that the cMET signaling is one of the most important pathways involving in tumorigenicity and metastasis of breast cancer cells^2^ and the key factor in this process is STAT3.^26^ The blocking of cMET expression caused the declining of STAT3 expression, although NPs loaded with DTX could somewhat be effective on decreasing the expression of STAT3 gene too. The dramatic declining of STAT3 gene expression occurred when combination therapy accompanied by targeted therapy. Similarly, regarding the other studied genes (MMP2, MMP9, VEGF, and IL8), targeted combination therapy as compared to passive combination therapy, had the highest impact on gene expression attenuation. Detailed comparisons represented that the combination therapy, either targeted or passive, had better effects on decreasing the gene expression of MMP2, MMP9 and VEGF vs. STAT3 and IL8. Moreover, gene expression of MMP9 was more affected by NPs loaded with DTX, while NPs loaded with cMET siRNA had more impacts on gene expression of IL8 and STAT3. Therefore, as each gene was especially affected by the different part of the combination therapy, it can be suggested that the application of both chemotherapeutic agent and siRNA may be beneficial for providing the maximum anti-cancerous effects.

## Conclusion

Our study was focused on usage of targeted combination therapy involving chitosan NPs, mucin1 APT, the chemotherapeutic agent DTX, and cMET siRNA. This is suggested as an effective treatment of mucin1^+^ metastatic breast cancer on the cell line level. The advantage of this combination, which can lead to more investigations on animal models, is the targeted delivery of drugs. This may consequently cause dose reduction and fewer cell toxicities. Moreover, the dual effects of chemotherapy and gene therapy can be accompanied by application of this suggested delivery system.

## Acknowledgments

This work was supported by the Drug Applied Research Center of Tabriz university of Medical Sciences [grant numbers 128/93].

## Ethical Issues

This study was approved by ethics committee at Tabriz University of Medical Sciences. The ethical code is TBZMED.REC.1394.413.

## Conflict of Interest

There is no conflict of interest to declare.
